# Caught between rights and realities: Nurses’ challenges with upholding patients’ rights in selected health facilities of South Africa

**DOI:** 10.4102/hsag.v31i0.3285

**Published:** 2026-03-06

**Authors:** Adolphina M. Thema, Fhumulani M. Mulaudzi, Ndivhaleni R. Lavhelani, Sinethemba Nyandeni, Maurine R. Musie

**Affiliations:** 1 Department of Nursing Science, Faculty of Health Sciences, University of Pretoria, Pretoria, South Africa

**Keywords:** nurses’ challenges, patients’ rights, patient safety, uphold, rights, violations

## Abstract

**Background:**

Nurses play a central role in upholding patients’ rights; yet in many health facilities, these rights are frequently compromised. Understanding these challenges is essential for developing effective improvement strategies and ensuring ethical, patients’ rights-centred care in health facilities.

**Aim:**

To explore and describe the challenges faced by nurses in upholding patients’ rights in selected health facilities within the Capricorn District, Limpopo province, South Africa.

**Setting:**

The study was conducted in four health facilities, including a tertiary hospital, district hospital, clinic, and healthcare centre.

**Methods:**

A qualitative, exploratory, descriptive, and contextual design was used. Data were collected through semi-structured interviews with 28 conveniently sampled nurses from different nursing categories. Data were analysed using Tesch’s open coding method.

**Results:**

Three themes with six subthemes were identified: (1) resource deficiencies as barriers hindering nurses’ ability to uphold patients’ rights; (2) nurses’ difficulties in upholding patients’ rights in health facilities; (3) infrastructure constraints affecting nurses’ ability to uphold patients’ rights in health facilities.

**Conclusion:**

The findings reveal that nurses’ ability to uphold patients’ rights is challenged by interrelated resources, patient-related, and infrastructure barriers. Addressing these challenges through adequate staffing, supply provision, infrastructure improvements, and supportive policies is critical for creating safe health facilities for patients.

**Contribution:**

This study provides evidence to assist policymakers, educators, and health administrators in developing sustainable policies that maintain professional ethics and comply with the constitutional requirement for equitable healthcare. It underscores the need for policy reforms, better resourcing, and supportive environments to strengthen nurses’ capacity for rights-based, ethical, and patient-centred care.

## Introduction

Upholding patients’ rights is a fundamental aspect of providing high-quality healthcare; however, the reality is that nurses frequently face challenges that undermine their capacity to uphold these rights. Challenges such as chronic shortages in nursing staff globally lead to high workloads, burnout, a lack of adherence to guidelines, and inadequate delivery of patient-centred care (Nesengani, Downing & Ten Ham-Baloyi 2025; Nsiah, Siakwa & Ninnoni 2020; World Health Organization 2025). Studies conducted in South Africa (SA) indicate that more than one-third (32.2%) of nurses experience various challenges in their occupation, with nearly half (46.1%) of them intending to leave their current job (Scheepers, Coetzee & Fourie 2025). Given these realities, it is important to understand nurses’ challenges in upholding patients’ rights in health facilities. Exploring and understanding these challenges will help formulate effective strategies to ensure that nurses are well prepared in their endeavour to uphold patients’ rights in health facilities (Thema, Mulaudzi & Lavhelani 2024).

The evolution of patients’ rights is anchored in international human rights instruments, which laid the groundwork for their recognition in healthcare. The Universal Declaration of Human Rights (UDHR) is the initial significant advancement in the establishment of patients’ rights (Mousavi et al. 2017). Patients’ rights are a fundamental aspect of human rights, promoting mutual respect between patients and health care providers while ensuring humane treatment in healthcare (Fouad, Abdelrahman & Mohamed 2020; World Health Organization 2020). Patients’ Rights Charter in Africa serves to promote and uphold patients’ rights in health facilities; but in most countries, patients’ rights are not upheld (Yarney et al. 2016). The persistent gap between policy and practice with regard to patients’ rights raises important questions about how these rights are upheld in everyday healthcare delivery.

Upholding patients’ rights has emerged as a fundamental principle in healthcare, providing a foundation for ethical conduct and professional responsibility. Healthcare facilities in SA should ensure that the services rendered uphold patients’ rights according to the Patients’ Rights Charter of SA (Thema & Sumbane 2022). These rights of patients include a right to a healthy and safe environment; participation in decision-making regarding patients’ health care; access to health care services; knowledge of health insurance or medical aid; choice of health services; informed consent; right to accept or refuse medical treatment; continuity of care; right to voice complaints about health services; referral for a second opinion; confidentiality and privacy; and the right to be treated by a named health care provider (Department of Health 1999).

Nurses are obligated to advocate for and uphold patients’ rights; however, several studies reveal that various challenges, such as shortage of resources and systemic inefficiencies, persistently hinder nurses’ ability to uphold patients’ rights (Nsiah et al. 2020). In Israel, medical professionals have exhibited hesitations and a lack of awareness in upholding patients’ rights (Sperling & Pikkel 2020:7). It is concerning that, despite universal recognition and publications of patients’ rights, nurses continue to encounter challenges in adequately upholding these rights.

A prevalent global challenge hindering nurses’ ability to uphold patients’ rights is the shortage of nursing professionals in health facilities. A survey revealed that sometimes, patients are being neglected in pain and, in some instances, left to die alone because of insufficient registered nurses on shifts (Brown 2024). Shortages in nursing staff lead to extended patient waiting times, and diminished access to care, thus violating patients’ right to access timely healthcare (Makua & Khunou 2022). Moreover, the right to confidentiality and privacy is often violated by overcrowding of patients and poor infrastructure, forcing patients to be examined or counselled in overcrowded consulting rooms (South African Human Rights Commission 2018). When nurses are unable to uphold patients’ rights as a result of these challenges, violations of these rights will be on the rise (Mutshatshi & Munyai 2022). As of 2025, limited research exists on nurses’ challenges in upholding patients’ rights in South Africa. Exploring these challenges can guide future studies and inform strategies to combat these challenges hindering ethical patient care.

To address the challenges nurses face in upholding patients’ rights, there is a need to set a foundation of improvement plans to eradicate these challenges. Policies and guidelines should be developed to ensure that patients’ rights are upheld without compromise. Consistently evaluating and contemplating ethical actions enables nurses to recognise areas for improvement with regard to maintaining patient rights (Wong et al. 2025). By exploring nurses’ challenges with upholding patients’ rights, health facilities can be made aware of their shortcomings and be able to improve on those challenges. This study therefore aims to explore and describe nurses’ challenges in upholding patients’ rights. The study was guided by the Donabedian’s framework alongside a Human Rights-Based Approach to Health (HRBAH) to carefully assess the manifestation of challenges encountered by nurses in upholding patients’ rights.

## Research aims and objectives

This study aims to explore and describe nurses’ challenges in upholding patients’ rights in the selected health facilities of the Capricorn District, Limpopo province, SA.

## Research methods and design

### Study design

This study employed a qualitative methodology with an exploratory, descriptive, and contextual design.

### Setting

The study was carried out in four health facilities in Capricorn District of the Limpopo province, SA, comprising a tertiary hospital, a district hospital, a clinic, and a community health centre. Capricorn district was selected based on the fact that it has the only two tertiary hospitals in the Limpopo province. There are other health facilities which also reflects the types of public health facilities found in Limpopo province, SA.

### Study population and sampling strategy

After obtaining ethical clearance and permission to conduct the study (Online Appendix 1), the researcher, introduced to the nursing staff by unit managers, recruited participants individually. Potential candidates were provided with an information sheet detailing the study and asked to give written consent (Online Appendix 2). The study population comprised nurses of all categories who were registered with the South African Nursing Council and employed at the selected health facilities of Capricorn District in Limpopo, South Africa, during 2023. Convenient non-probability sampling was used, including nurses available during data collection.This approach proved suitable because of the variations in facility size, service scope, and staff makeup, enabling the inclusion of nurses present during their shifts without interrupting service delivery. The objective was to acquire varied, contextually rich insights on the extent to which patients’ rights are being upheld in the selected health facilities rather than statistical representativeness.

A total of 28 nurses participated in the study, who were selected from the four health facilities within the Capricorn District. Participants represented diverse professional categories registered with the South African Nursing Council including professional nurses (RN), enrolled nurses (EN), enrolled nursing assistants (ENA), and operational managers (OPM), with ages ranging from 25 to 58 years. Those unavailable during data collection were excluded from the study. The inclusion of a diverse group was intentional to capture varied experiences of patient-rights compliance across different facility levels and scopes of practice, thereby enriching the depth and contextual understanding of the phenomenon. Participants were selected through convenient non-probability sampling based on their availability and willingness to participate. Data collection and analysis were conducted concurrently, and recruitment ceased when no new information emerged, indicating data saturation at 28 participants.

### Data collection

An interview guide with open-ended questions (Online Appendix 3) was developed from relevant literature and study objectives, using the Donabedian framework to assess nurses’ challenges with upholding patients’ rights in the selected health facilities, with emphasis on the structure and process domains. Its validity was tested by conducting a pilot study on four participants from the selected health facilities to ensure clarity, relevance, comprehensiveness, and it was reviewed by the University’s research ethics committee. Iterative revisions were made to the interview guide based on feedback and pilot data. The researcher received training on data collection processes from the supervisors to conduct the interviews effectively. Those who participated in the pilot study were excluded from the main study, and the findings from the pilot were also not included in the final analysis.

Unit managers assisted with the recruitment of participants by sharing information sheets with eligible staff, who then volunteered to participate. The interviews were semi-structured, held in English to avoid leading questions, interviewer bias and conducted in private rooms within the selected health facilities in the Capricorn District of Limpopo province, SA. Participants received information about the study and provided written consent before taking part.

Data were collected using the interview guide through one-on-one semi-structured interviews, beginning with a central question and supported by probing questions. To protect confidentiality and reduce response bias, no names were recorded. Interviews were conducted in quiet spaces within the selected health facilities, lasting 15 min–50 min. Using the central question, ‘Please explain the challenges you experience with upholding Patients’ Rights when nursing patients in this health facility’, the researcher explored the structure and process domain of the Donabedian framework.

Probing questions were used during the interviews to obtain detailed insights into nurses’ challenges in upholding patients’ rights, with follow-up questions asked for clarity and deeper understanding (Polit & Beck 2020). Data collection was supported by field notes and audio recordings to enhance validity and confirmability. Participants gave permission for recordings and were informed of their purpose, while descriptive field notes were taken throughout. All interviews were transcribed verbatim. Data saturation determined the sample size.

### Data analysis

Data from the semi-structured, in-depth interviews were analysed using Tesch’s eight-step open coding method of qualitative data analysis (Creswell 2016). Tesch’s method was selected for its systematic and adaptable analytical framework, which supports inductive, data-driven exploration of complex and context-dependent phenomena such as nurses’ challenges in upholding patients’ rights. The researcher reviewed all transcripts to grasp the whole context, and subsequently choosing one transcript at a time to determine the underlying meanings and themes that emerged. Similar themes were categorised and condensed into codes, which were subsequently applied to all transcripts. Codes were carefully refined and categorised, leading to the development of principal themes and subthemes that encapsulated the core of participants’ experiences.

The researcher engaged in an iterative process, alternating between data collection and analysis to systematically organise (using ATLAS.ti software), interpret, and make sense of participants’ experiences within their contextual settings. An independent coder was sent 28 transcripts for analysis, who also employed Tesch’s eight-step open coding method. A consensus meeting was held between the researcher and co-coder; thereafter, both analyses were compared to evaluate consistency, ensure depth, and identify any overlooked themes or subthemes. Three major themes emerged (see Table 1 for themes and subthemes).

**TABLE 1:** Themes and subthemes.

Themes	Subthemes
1. Resource deficiencies as barriers hindering nurses’ ability to uphold patients’ rights in health facilities.	1.1. Inadequate human resource in health facilities. 1.2. Non-human resource limitations in health facilities.
2. Nurses’ difficulties in upholding patients’ rights in health facilities.	2.1. Patient-related factors limiting patients’ rights being upheld by nurses. 2.2. Execution of non-nursing duties by nurses in health facilities.
3. Infrastructure constraints affecting nurses’ ability to uphold patients’ rights in health facilities.	3.1. Interruptions in essential services compromising nurses’ ability to uphold patients’ rights. 3.2. Health facility layout limitations hindering nurses’ ability to uphold patients’ rights.

### Measures to ensure trustworthiness

The study’s author is a licensed nurse without any supervisory or professional ties to the participants, hence reducing power disparities and potential bias in response. Reflexivity was upheld by continuous contemplation of personal assumptions and the utilisation of a reflexive journal to guarantee that data interpretation remained participant-centred and credible. To enhance credibility and minimise confirmation bias, 28 transcripts were submitted via email to an independent coder, who also applied Tesch’s eight-step method of data analysis. The study included techniques like prolonged participant involvement, triangulation across many nursing categories, and independent coding followed by consensus discussion to guarantee internal validity (credibility). To further strengthen confirmability and dependability, a consensus meeting was held between the researcher and the independent coder to review and finalise the themes and subthemes.

Transferability was ensured by providing detailed descriptions of the study context, participants, and research process, enabling readers to assess the applicability of findings in similar settings. A thorough audit trail improved transparency, and member verification was also employed to ensure that the interpretations appropriately represented the opinions of the participants. Convenient non-probability sampling of nurses from various professional categories and facilities, along with detailed, contextual descriptions of the environment and participants, supported external validity (transferability), allowing readers to evaluate the findings’ generalisability to different situations.

## Results

The study explored nurses’ challenges with upholding patients’ rights in health facilities. Analysis of the data revealed three main themes with a total of six subthemes, highlighting a complex interplay of challenges that affect nurses’ ability to provide care that respects patients’ rights. These challenges ranged from resource deficiencies and patient-related behaviours to infrastructural constraints. Presenting the findings thematically allows a clear understanding of how these challenges manifest in daily nursing practice (see Table 1).

### Theme 1: Resource deficiencies as barriers hindering nurses’ ability to uphold patients’ rights in health facilities

The first theme emphasises the critical role of sufficient resources in enabling nurses to uphold patients’ rights. This theme comprises two subthemes:

#### Subtheme 1.1: Inadequate human resource in health facilities

Participants consistently reported that staffing shortages significantly limited their ability to deliver optimal care centred on patients’ rights. Nurses described situations in which they lacked sufficient time to educate or attend to patients properly, or had to compromise hygiene standards because of limited cleaning staff. This was evidenced by the following statements from the participants:

‘The challenges it can be shortage of staff, sometimes we do not have enough time to explain to the patient because we have lot of things to do. Yes, so we do not have, we cannot have time to speak and explain to the patient properly.’ (Participant no 9, RN, tertiary hospital)

‘With clean environment, the challenge is shortage of cleaners because you find that we are working with one cleaner per shift so we have challenges, you find that you are forced to work in a dirty environment because you are waiting for a cleaner who is working on the other side and you must also work.’ (Participant no 10, EN, tertiary hospital)

‘We are short-staffed as nurses and the patients are many.’ (Participant no 27, EN, community health centre)

The findings demonstrate that inadequate human resources, including both clinical and support staff, significantly undermines nurses’ capacity to uphold patients’ rights. Staff shortages not only increase workloads and time pressures but also compromise patient education, communication, and environmental hygiene. This highlights how insufficient human resources directly hinder the provision of quality, rights-based care, leaving both patients and nurses vulnerable within the healthcare system.

#### Subtheme 1.2: Non-human resource limitations in health facilities

Shortages of equipment, medications, and other essential supplies were also highlighted as challenges hindering the ability of nurses to uphold patients’ rights. Nurses described how these limitations delayed procedures, increased patient stays, and compromised care quality. This was expressed by the participants with the following statements:

‘The equipment as well, that’s where the challenge is there most of the time, you find that most of the time the patient has been cancelled in the theatre due to some instrument not being available, so those are the kind of the challenges that we face, hence some will end up having a long stay or patient in the ward.’ (Participant no 12, OPM, tertiary hospital)

‘There is no medication.’ (Participant no 23, EN, district hospital)

‘Sometimes maybe we have got shortage of linen. We do not have hot water in the hospital. Like the patients have got the right to complain about this.’ (Participant no 1, ENA, district hospital)

The shortage of essential non-human resources, such as medical equipment, medications, linen, and even basic amenities like hot water, was found to compromise patients’ comfort, safety, and rights. These deficiencies often delay treatment, prolong hospital stays, and create conditions for patient dissatisfaction. The findings underscore that without adequate material resources, nurses’ ability to provide dignified, rights-based care remains severely constrained.

### Theme 2: Nurses’ difficulties in upholding patients’ rights in health facilities

The second theme reflects practical challenges in nursing practice arising from patient behaviour and additional non-nursing responsibilities. It includes two subthemes:

#### Subtheme 2.1: Patient-related factors limiting patients’ rights being upheld by nurses

Participants emphasised that patient behaviours, such as missed appointments, non-compliance with hygiene protocols, and aggression, limited their ability to provide care that upheld patients’ rights. This was revealed when the participants said the following:

‘Here in maternity ward, we see the patient and do what we do to her and you give her return date but they do not honor their return date, it seems as if it’s a joke to them and you are just putting it on just for the sake of putting it on.’ (Participant no 4, RN, community health centre)

‘When they eat things, they will and throw them on the floor … they sometimes just vomit and just leave everything there instead of using a bucket.’ (Participant no 8, ENA, tertiary hospital)

‘Most patients come with aggressive behavior and attitude for nurses so it makes us difficult to comply with patients’ rights especially because they come having no intention to listen to us and we end up not doing the correct things because of that.’ (Participant no 10, EN, tertiary hospital)

Patient-related factors, including missed appointments, disregard for hygiene protocols, and aggressive behaviours, emerged as significant challenges hindering nurses’ ability to respect and uphold patients’ rights. Such behaviours disrupted care processes, strained nurse-patient relationships, and, at times, forced nurses to compromise on best practices. These findings highlight that while patients are entitled to their rights, their active participation and cooperation are equally crucial in ensuring the realisation of those rights within healthcare facilities.

#### Subtheme 2.2: Execution of non-nursing duties by nurses in health facilities

Nurses also reported performing non-nursing tasks as a result of staff shortages, which limited time for patient care:

‘We have shortage of cleaners so if one cleaner is busy that side, I have to now perform her duties of cleaning first before I start with my nursing routine to save time.’ (Participant no 18, RN, community health centre).

‘Sometimes we clean and take the dustbins out because we do not have enough cleaners in the hospital, so if they are sick or they are still busy we take over.’ (Participant no 25, RN, district hospital)

‘Because we do not have a messenger, we send one nurse to run errands and remain working for them so the time is limited.’ (Participant no 12, OPM, tertiary hospital)

The diversion of nurses to non-nursing duties, such as cleaning, waste disposal, and running errands, significantly reduced the time available for patient care and undermined their ability to uphold patients’ rights. This issue of role overlaps not only diluted the professional function of nurses but also created conditions where essential nursing responsibilities were delayed or neglected. Addressing these gaps through the provision of adequate support staff is therefore critical to safeguarding patients’ rights and ensuring that nurses can focus on their core professional duties.

### Theme 3: Infrastructure constraints affecting nurses’ ability to uphold patients’ rights in health facilities

The third theme addresses infrastructural barriers that compromise the delivery of rights-based care. It comprises two subthemes:

#### Subtheme 3.1: Interruptions in essential services compromising nurses’ ability to uphold patients’ rights

Participants reported that interruptions in essential utilities, such as water and electricity, directly affected patient care, safety, and hygiene. These interruptions made it difficult for nurses to respect and uphold patients’ right to a safe and healthy environment. This was evidenced by participants saying the following:

‘We have water cuts affecting us with making sure that the patients are in a clean environment, and also they themselves need water to bath so without water, it’s difficult to comply with patients’ rights.’ (Participant no 22, OPM, district hospital)

‘With the constant electricity outages, we nurse patients in the dark and use our cellphones to light up the room, that is not safe at all.’ (Participant no 17, OPM, district hospital)

‘Because of power outage, patients also panic since we do not have backup supply of electricity here.’ (Participant no 13, OPM, tertiary hospital)

Interruptions in essential services such as water and electricity directly threatened the fulfilment of patients’ rights to safety, dignity, and a clean environment. These disruptions not only jeopardised infection prevention and basic patient care but also caused anxiety and discomfort among patients. The findings emphasise that reliable infrastructure and uninterrupted utility supply are foundational to enabling nurses to uphold patients’ rights effectively.

#### Subtheme 3.2: Health facility layout limitations hindering nurses’ ability to uphold patients’ rights

Inadequate infrastructure, difficulties in maintaining confidentiality, a lack of privacy and patient comfort, were identified as major challenges encountered by nurses to uphold patients’ rights. The participants said the following:

‘I cannot close the windows, because we need fresh air, the windows do not have the curtains, no mobile screens, so patient do not feel comfortable.’ (Participant no 5, RN, clinic)

‘We do not have like a room, we do not even have a room, a psychology room we do not have it, we end up talking in front of other patients even if we try to keep the voice down.’ (Participant no 7, RN, tertiary hospital)

‘It is a challenge because there’s no private room to discuss private issues, you have to say it in front of others.’ (Participant no 23, EN, district hospital)

Inadequate infrastructure, including the lack of private consultation spaces, curtains, and mobile patient privacy screens, were found to compromise patient privacy, comfort, and confidentiality. Such limitations not only diminished the quality of care but also left patients feeling exposed and vulnerable in sensitive situations. These findings highlight that a supportive physical environment is essential to ensuring patients’ rights are respected and to fostering trust between patients and healthcare providers.

## Discussion

This study aimed to explore the multifaceted challenges nurses encounter in upholding patients’ rights within health facilities. Donabedian’s framework consisting of structure, process, outcome domain together with a HRBAH served as the foundation for the examination of the themes and subthemes (Ayanian & Markel 2016; Mahomed et al. 2020). Resource-related issues, such as staff, equipment, and facility infrastructure constraints, were brought to light by the structural domain. The investigation of nurse-patient interactions was guided by the process domain, which also helped to discover patient-related aspects including aggressive behaviours and non-compliance. The outcome domain shed light on the effects of these difficulties on patients’ rights, such as diminished comfort, hygienic conditions, and treatment quality. Integrating HRBAH made guaranteed that the conversation stayed focused on patients’ rights, highlighting both practical and ethical issues.

The abovementioned approaches (Donabedian’s framework and HRBAH) provided a structured lens through which one can evaluate the data, allowing for a clear presentation of the themes and subthemes that reflect the multifaceted problems nurses encounter when upholding patients’ rights. ‘Resource deficiencies as barriers hindering nurses’ ability to uphold patients’ rights in health facilities’, is the first theme identified in this study.

The first theme highlights significant resource deficiencies, both human and non-human, as primary challenges to nurses’ ability in upholding patients’ rights. Participants reported that understaffing and shortages of essential supplies compromised their ability to provide adequate care. These findings align with existing literature, which indicates that inadequate staffing and resource shortages are prevalent issues in healthcare settings, leading to increased workloads and diminished care quality (Nesengani et al. 2025; World Health Organization 2025). Similarly, a study carried out in the province of Limpopo, SA, found that rural areas lack human resources, which makes it difficult to integrate and use a complete approach in the administration and delivery of quality health services (Mutshatshi & Munyai 2022). Overall, shortages in both human and non-human resources greatly impede nurses’ capacity to uphold patients’ rights, adversely impacting treatment quality and patient comfort. Beyond these resource limitations, nurses also encounter patient-related challenges that further complicate the realisation of patients’ rights.

The second theme therefore examines issues such as non-compliance with treatment protocols and aggressive behaviours, which emerged as additional challenges within healthcare facilities. The participants expressed challenges in maintaining a safe and healthy environment when patients failed to adhere to hygiene standards, follow-up appointments or exhibited disruptive behaviours. This resonates with a previous study that has documented how patient behaviours can hinder the provision of ethical care, emphasising the need for effective communication and behavioural management strategies (Nsiah et al. 2020). These findings underscore that nurses’ capacity to uphold patients’ rights is influenced not only by institutional and professional variables but also by patient behaviours, which may jeopardise patient safety and the quality of care.

Nurses furthermore expressed the burden of performing non-nursing duties, which resulted in them neglecting their core nursing duties and hindering their ability to uphold patients’ rights when caring for patients in health facilities. These findings are similar to those of a Canadian survey, where 33 000 nurses acknowledged that they compromise professional nursing when they neglect important tasks due to excessive workloads (MacPhee, Dahinten & Havaei 2017). It is evident that the need to carry out non-nursing tasks severely limited nurses’ capacity to uphold patients’ rights, making daily nursing care even more difficult.

The third theme addresses infrastructural limitations, including unreliable utilities such as water supply, power supply and inadequate physical environments, which impede nurses’ ability to uphold patients’ rights. Participants noted that power outages and water shortages and a lack of privacy in the health facilities compromised patient safety and dignity. These findings are consistent with research highlighting how systemic issues, such as infrastructure deficiencies, contribute to ethical challenges in clinical settings (Scheepers et al. 2025). According to National Institute For Communicable Diseases (2023), the provision of quality healthcare is contingent upon a safe and hygienic environment, supported by reliable access to clean running water for both patients and healthcare staff. Such conditions are not only essential for effective care delivery but also form a fundamental component of upholding patients’ rights to dignity, safety, and quality treatment.

### Implications for nursing practice

The study’s findings suggest several implications for nursing practice. Firstly, addressing resource deficiencies through adequate staffing and supply management is crucial for enabling nurses to provide care that respects patients’ rights. Secondly, training programmes focusing on managing patient behaviours and enhancing communication skills can equip nurses with strategies to navigate challenging patient interactions. Lastly, advocating for infrastructure improvements, such as reliable utilities and private spaces, is essential for creating an environment conducive to ethical care delivery.

### Strengths and limitations of the study

This study is strengthened by its use of Donabedian’s framework and the HRBAH, which ensured that the findings were grounded in a systematic evaluation of healthcare structures, processes, and outcomes while centring patients’ rights. By combining these frameworks with a qualitative, contextual design, the study provides rigorous, theory-informed, and actionable insights into the challenges nurses face in upholding patients’ rights, supporting evidence-based policy and practice improvements. Additionally, the study adds to a neglected field of study by providing important data to guide future training, policy, and practice activities. Additionally, steps to ensure reliability, such as independent coding, consensus meetings, pilot testing of the interview guide, and the use of credibility, dependability, confirmability, and transferability criteria, increased the study’s rigour.

However, the study does have a few limitations. The results may not apply to other areas or healthcare systems because they are context-specific. As the study relied on self-reported interview data, the findings may have been influenced by participants’ subjective interpretations and their willingness to disclose information fully. Notwithstanding these limitations, the study offers valuable insights into the challenges that nurses encounter in upholding patients’ rights and lays the groundwork for further investigation on this topic. Future research should aim to include a more diverse sample and employ longitudinal designs to assess the impact of interventions aimed at overcoming the identified challenges.

## Conclusion

This study reveals that although nurses play a crucial role in protecting and advocating for patients’ rights, systemic, institutional, and contextual obstacles limit their capacity to do so. The results highlight the need for structural change, adequate resources, and encouraging organisational cultures that foster moral and patient-centred treatment in order to improve patients’ rights compliance. Transforming policy intent into everyday clinical reality will be made easier by enhancing nurses’ competence through ongoing professional development, moral leadership, and supportive work environments. The study emphasises how urgently policymakers, health managers, and nursing educators must work together to implement patient rights as a lived experience rather than a legal obligation. By illuminating the lived experiences of nurses across multiple levels of care, this research contributes to the broader discourse on human rights in healthcare and offers evidence to guide policy, training, and institutional transformation.
